# Dynamic Remodeling of the Zona Pellucida: Implications for Oocyte Competence and Assisted Reproduction

**DOI:** 10.3390/ijms262211108

**Published:** 2025-11-17

**Authors:** Daniel de la Fuente, Michela Prestianni, Paula Navarrete-López, Cristina García-Merino, Miriam Balastegui-Alarcón, Pilar Soria, Manuel Avilés, Dimitrios Rizos, Alfonso Gutiérrez-Adan

**Affiliations:** 1 Department of Animal Reproduction, INIA-CSIC, 28040 Madrid, Spain; dandel02@ucm.es (D.d.l.F.); michela.prestianni@inia.csic.es (M.P.); paulanavarrete116@gmail.com (P.N.-L.); cristina.garcia@inia.csic.es (C.G.-M.); drizos@inia.csic.es (D.R.); 2 Fiv Center Clinic, 28023 Madrid, Spain; 3 Department of Cell Biology and Histology, Instituto Murciano de Investigación Biosanitaria Pascual Parrilla, Universidad de Murcia, 30120 Murcia, Spain; miriam.balastegui@um.es (M.B.-A.); p.soriamonzo@um.es (P.S.); maviles@um.es (M.A.)

**Keywords:** assisted reproductive techniques, fertilization, hatching, maturation, ovastacin, oviductin, sperm binding, zona pellucida glycoproteins

## Abstract

The zona pellucida (ZP) is a glycoprotein-rich extracellular matrix essential for fertilization, early embryonic development, and implantation. Beyond its core functions, the ZP undergoes dynamic remodeling during oocyte maturation, involving regulated synthesis, assembly, and conformational changes. This complex and tightly controlled process ensures the biomechanical integrity of the oocyte, providing both protection and selective permeability essential for reproductive success. Oviductal glycoprotein 1 (OVGP1; oviductin) integrates into the ZP, modulating pore size, glycan composition, and structural homogeneity. This glycoprotein establishes a species-specific barrier that prevents polyspermy and fine-tunes sperm–oocyte interactions. Recent evidence suggests that OVGP1 not only contributes to ZP stabilization but also influences sperm capacitation, acrosome reaction, and early zygotic signaling, thereby linking oviductal physiology to gamete compatibility. Exploiting ZP remodeling in assisted reproductive technologies (ART) offers new opportunities to enhance fertilization efficiency, embryo quality, and implantation success, including through assisted hatching or modulating ZP properties to overcome fertility limitations. Moreover, advances in proteomic and glycomic profiling of the ZP are enabling the identification of novel biomarkers of oocyte competence and reproductive potential. These advances provide critical insights into species-specific reproductive mechanisms and pave the way for improved ART protocols and fertility preservation strategies.

## 1. Introduction

The zona pellucida (ZP) is a glycoprotein-rich extracellular matrix that surrounds the mammalian oocyte and it is essential for oogenesis, fertilization, and early embryonic development [1]. It is mainly composed of four glycoproteins: ZP1, ZP2, ZP3, and ZP4, whose structural integrity relies on non-covalent interactions. Each glycoprotein contains a linker region and a ZP module with two conserved subdomains, ZP-N and ZP-C, that regulate activity [2]. Its three-dimensional structure resembles an Ig-like fold [3]. The ZP pays a critical role in species-specific fertilization by mediating sperm-oocyte binding. Previous research has examined whether the sperm from distant mammalian orders (Carnivora, Primates, and Rodentia) can penetrate bovine oocytes, as well as the influence of bovine oviductal fluid (OF) and species-specific oviductal glycoprotein (OVGP1 or oviductin) from bovine, murine, or human sources in modulating the species-specificity of bovine and murine oocytes. Sperm from all the species were able to penetrate intact bovine ovarian oocytes and form hybrid embryos. However, exposure to OF or bovine, murine, or human OVGP1, conferred the ZP species-specificity, allowing only sperm of the corresponding species to penetrate the oocyte, regardless of the ZP’s origin [4]. Glycosylation and microstructural analyses revealed that OVGP1 covers the pores of the ZP and its glycosylation determines sperm specificity [4].

Exposure of the oocyte to OVGP1 within the oviduct appears to refine ZP maturation by adjusting its porosity, mechanical stability, and carbohydrate presentation, all of which determine species-restricted sperm binding and penetration. By integrating into the ZP matrix, OVGP1 reorganizes its filamentous structure and modulates its sialylation. This remodeling step within the oviduct is therefore central to the complete and specific acquisition of fertilization competence. Its role in shaping the biomechanical properties of the ZP provides a mechanistic framework to explain how oviductal factors control sperm–oocyte interaction and early embryo development. As ZP maturation influences both sperm–oocyte interaction and embryonic competence, understanding the contribution of OVGP1 is essential for refining assisted reproductive technologies. Future research should explore how the oviductal environment and OVGP1-mediated modifications can be emulated or supported during in vitro procedures. Such advances could improve fertilization efficiency, enhance embryo quality, and help bridge the current gap between physiological and in vitro conditions in human ART.

## 2. Zona Pellucida Composition and Its Evolutionary Origin

The ZP is an extracellular matrix that surrounds all mammalian oocytes, eggs, and early embryos and plays critical roles during oogenesis, fertilization, and preimplantation development. The ZP is mainly composed of three or four glycosylated proteins (ZP1–ZP4) that are synthesized, processed, secreted, and assembled into long, cross-linked fibrils by growing oocytes. ZP proteins have an immunoglobulin-like three-dimensional structure and a ZP domain consisting of two subdomains, ZP-N and ZP-C, with ZP-N of ZP2 and ZP3 required for fibril assembly [2]. A ZP2–ZP3 dimer is located periodically along ZP fibrils and is cross-linked by ZP1, a protein with a proline-rich N terminus. Fibrils in the inner and outer regions of the ZP are oriented perpendicular and parallel to the oolemma, respectively, giving the ZP a multilayered appearance. Upon fertilization, modification of ZP2 and ZP3 results in changes in the ZP’s physical and biological properties that have important consequences. Certain structural features of ZP proteins suggest that they may be amyloid-like proteins. Following the fertilization of an egg by a single sperm, the egg coat, or ZP, hardens, and polyspermy is irreversibly blocked. These events are associated with the cleavage of the N-terminal region (NTR) of glycoprotein ZP2, a major subunit of ZP filaments. ZP2 processing is thought to inactivate sperm binding to the ZP, but its molecular consequences and connection with ZP hardening remain unknown. Biochemical and structural studies show that cleavage of ZP2 triggers its oligomerization. Moreover, the structure of a native vertebrate egg coat filament, combined with AlphaFold predictions of human ZP polymers, reveals that two protofilaments consisting of type I (ZP3) and type II (ZP1/ZP2/ZP4) components interlock into a left-handed double helix from which the NTRs of type II subunits protrude [5]. Together, these data suggest that oligomerization of cleaved ZP2 NTRs extensively cross-links ZP filaments, rigidifying the egg coat and making it physically impenetrable to sperm.

**Figure 1:**
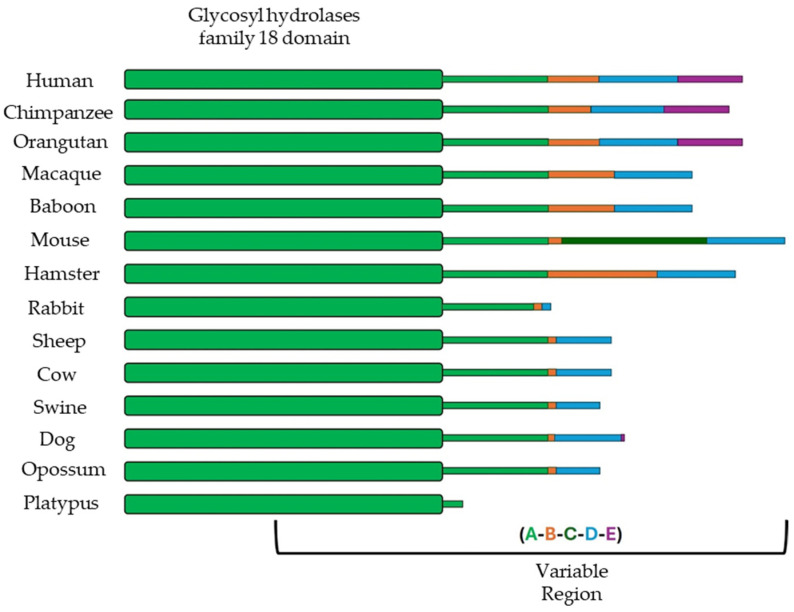
Schematic representation of the regions (A, B, C, D, and E) of the oviductal glycoprotein 1 (OVGP1) in different mammalian species. Figure modified from [22].

ZP composition differs among species: in mice, it consists of ZP1, ZP2 (ZPA), and ZP3 (ZPC), while in cows, pigs, and dogs it is formed by ZP2 (ZPA), ZP4 (ZPB), and ZP3 (ZPC) [6]. Humans, rats, horses, cats, monkeys, rabbits, and hamsters express all four proteins [7,8] (Table 1; Figure 1). ZP thickness also varies, being ~16 μm in cows, 18 μm in pigs, 12 μm in humans, and 8 μm in mice [9,10,11], with pore sizes ranging from 50 to 182 nm [12]. Despite high conservation, the ZP exhibits structural variability that contributes to species-specific fertilization. In many mammals, it initially acts as a non-specific barrier until it interacts with oviductal fluid (OF), particularly OVGP1. Nevertheless, its conserved roles include sperm binding and selection, induction of the acrosome reaction, prevention of polyspermy, and protection of the oocyte and embryo [4]. These functions are both mechanical and immunological. The ZP also regulates embryo hatching and implantation, after which it is shed.

**Figure 2:**
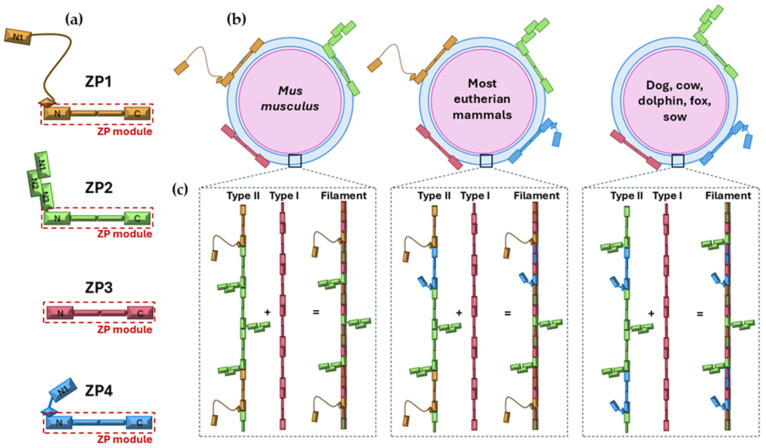
Molecular architecture of zona pellucida (ZP) proteins, ZP composition in different eutherian mammals and protofilaments and filaments organization. Schematic representation of the four ZP glycoproteins (ZP1, ZP2, ZP3, and ZP4) highlighting the ZP module (red box) and showing the differences in their N-terminal region (**a**). ZP composition in representative mammalian groups: *Mus musculus*, with ZP4 pseudogenized; most eutherian mammals (humans, the genus Mus, rat, rabbit and hamster), expressing all 4 ZP proteins, and dog, cow, dolphin, fox, and sow, lacking ZP1 (**b**). Assembly of type I protofilaments (composed of ZP3 only) and type II protofilaments (formed by variable combinations of ZP1, ZP2, and/or ZP4) into filaments that constitute the ZP matrix (**c**). Figure modified from [5].

Structurally, the ZP is a heterogeneous matrix. ZP2 and ZP3 polymerize through their ZP-N subdomains, while ZP1 acts as a molecular crosslinker via its proline-rich N-terminal extension, stabilizing and providing flexibility to the network [5,23]. These glycoproteins are secreted mainly by the oocyte, though granulosa cells contribute in some species, including humans [11,14]. They assemble into a fibrous network that provides elasticity, birefringence, and porosity, organized into asymmetric outer, intermediate, and inner layers visible under polarized light microscopy [24]. In the mature ZP, filaments form two intertwined protofilaments arrange in a left-handed helix: type I subunits (ZP3) and type II components (ZP1, ZP2, ZP4), replacing the previously proposed parallel–perpendicular model (Figure 2). After fertilization, N-terminal cleavage of ZP2 initiates oligomerization and cross-linking, stiffening the ZP and establishing a permanent polyspermy block in most mammals [5,25]. However, in lagomorphs such as rabbits, polyspermy prevention occurs at the oolemma, with multiple spermatozoa present in the perivitelline space [26,27].

The murine model has been central to functional studies of ZP proteins. ZP1-deficient female mice remain fertile but exhibit reduced reproductive success, whereas ZP2- or ZP3-null mice are infertile and produce eggs without a ZP [28]. These mice also fail to establish gap junctions with follicular cells, impairing oocyte-follicle communication and resulting in infertility due to absent growing oocytes and multilayered follicles [29], although fertilization can be rescued by ICSI. Comparable effects have been reported in humans: mutations in ZP genes cause abnormal or absent ZPs, impaired oogenesis, and infertility [30,31,32,33,34,35,36]. For instance, hZP4 variants are associated with thin or irregular ZPs, suggesting defective assembly [37]. The ZP presence is conserved across vertebrates, with homologous envelopes in fish, amphibians, and reptiles that share structural and functional features, particularly in sperm-egg interactions. Phylogenetic analyses show that ZP genes underwent duplication and divergence. This leads to the emergence of distinct ZP1–ZP4 proteins in mammals as species-specific adaptations for fertilization and polyspermy prevention. As noted above, while humans retain all four glycoproteins, mice lack ZP4 and cows, dogs, and pigs lack ZP1 [14,38]. Missing genes often persist as pseudogenes, with functional compensation by others. Conservation of ZP1–ZP3 ranges between 60 and 98% [39]. Sequence comparisons with human ZP proteins classify species into four groups: Group I (trout, 33%), Group II (frog, chicken, possum, 43–51%), Group III (mouse, rat, cow, pig, dog, 64–69%), and Group IV (macaques, chimpanzees, 93–99%), with >40% identity generally indicating functional conservation [40].

Gene subfamilies include ZPA, ZPB, ZPC, and ZPX [41]. Other analyses identify six ancestral subfamilies: ZPA/ZP2, ZPB/ZP4, ZPC/ZP3, ZP1, ZPD, and ZPAX [6]. ZPD is restricted to some bird and amphibian lineages, while in monotremes like platypus, eight subfamilies exist (ZP1, ZP2, ZP3a, ZP3b, ZP3-2, ZP4, ZPAX, ZPY) [20]. In marsupials, ZP3 diversified into three subfamilies (ZP3-1a, ZP3-1b, ZP3-1c) [14]. These likely arose from duplications in a common ancestor of marsupials and placentals. Subsequent simplification in placental mammals led to the loss of most subfamilies, retaining only one functional ZP3, possibly reflecting adaptations to internal fertilization.

Traditionally, ZP3 has been considered the primary sperm receptor, responsible for mediating binding of capacitated sperm, with ZP2 acting as a secondary receptor for reacted sperm [42]. However, recent studies indicate that ZP2 also binds unreacted sperm through its N-terminal domain, triggering conformational changes that block polyspermy [5]. In humans, hZP2 alone can induce sperm binding and acrosome reaction, while hZP1 also contributes. Current proteomic and transcriptomic approaches are expanding our understanding of ZP proteins [14]. Most placental mammals, including humans, hamsters, rabbits, and cats, lack ZPY, ZPAX, and two ZP3 subfamilies, retaining only four [7,8,43,44]. Some species, like humans, chimpanzees, and cows, retain ZPAX pseudogenes, while others, including dogs, cows, and rodents, show pseudogenization of ZP1 or ZP4 [14,16,43].

## 3. Zona Pellucida Maturation

### 3.1. Zona Pellucida Formation During Ovarian Development

During follicular maturation, granulosa cells differentiate and acquire the ability to synthesize specific ZP glycoproteins. The ovarian microenvironment is enriched in signaling molecules and growth factors. It strongly influences granulosa cell function and ZP formation. This dual origin highlights the interplay between oocytes and somatic cells in ZP formation, which is essential for fertilization and for early development. As noted, ZP thickness varies among species, for instance, ~6 µm in mice and ~20 µm in humans [30]. ZP formation begins early in folliculogenesis, when proteins secreted by the oocyte assemble into a fibrillar matrix that thickens as the oocyte matures [11,44]. Although ZP maturation is primarily driven by the oocyte, some studies suggest that cumulus cells also contribute [11,14]. Communication between the oocyte and cumulus cells through gap junctions across the ZP has been documented in several species, including humans, monkeys, cows, pigs, dogs, and rabbits [45,46,47]. This bidirectional signaling ensures coordinated development of both cumulus cells and ZP. During oocyte maturation, cumulus expansion occurs in parallel with ZP thickening and increased birefringence (Table 2).

**Figure 3:**
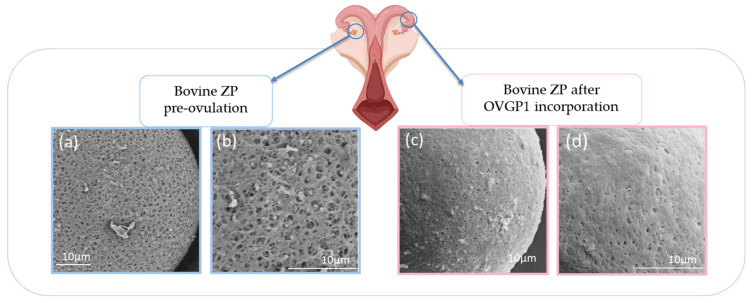
(Adapted from [4]). Bovine ZP changes during oocyte maturation. Bovine in vitro matured (IVM) oocytes. High magnification reveals the ultrastructural characteristics of the ZP’s pores on the oocytes without bOVGP1 (**a**,**b**) and with bOVGP1 (**c**,**d**). Scale bars = 10 μm.

Oocyte maturation involves structural ZP changes synchronized with nuclear and cytoplasmic events. This changes ensure meiotic resumption, fertilization, and embryonic development. In bovine oocytes, cumulus–oocyte junction pores narrow during maturation [49]. In humans, immature ZPs appear smooth and non-porous, whereas mature ZPs become rougher and reticulated [50]. In pigs, the immature ZP is porous and loose, but becomes denser and more mesh-like upon maturation [50]. In contrast, equine ZPs remain compact with small pores regardless of maturity, highlighting interspecies differences. In pigs, incomplete ZP maturation during IVF likely contributes to high polyspermy rates, a defect partially corrected by OF exposure [51]. In cattle, both pore number and size correlate with sperm binding and penetration efficiency, serving as indicators of oocyte quality [52]. At ovulation, the ZP undergoes dynamic remodeling of its glycoprotein composition and architecture [53]. These changes, mediated by molecular signals and cellular interactions, involve rearrangements in glycoprotein distribution that influence both ZP structure and its role in sperm–egg recognition (Figure 3).

### 3.2. Synthesis and Polymerization of ZP Proteins

During folliculogenesis, oocytes secrete ZP glycoproteins. Granulosa cells also contribute, creating a coordinated assembly process. ZP subunits co-polymerize into a two-protofilament filament: ZP3 provides the scaffold, ZP1 cross-links filaments via disulfide bonds, and ZP2, once cleaved after fertilization, oligomerizes to harden the matrix and block polyspermy [5]. ZP4, if present, supports fibril stabilization. ZP formation is regulated by hormonal signals, particularly FSH and estrogen, which control ZP gene expression. Altered expression or mutations in these genes may lead to infertility by disrupting ZP assembly [34].

### 3.3. Fertilization and ZP Modifications

Fertilization is the fusion of a haploid oocyte and a haploid sperm to form a diploid zygote. This process involves a series of cellular and biochemical events: the sperm acrosome reaction, recognition, binding, and penetration of the ZP, and fusion with the oolemma [54]. The ZP is not directly involved in this last step. First, sperm undergo the acrosome reaction, in which the plasma membrane fuses with the outer acrosomal membrane, releasing hydrolytic enzymes and exposing previously hidden surface ligands [55]. Both acrosome-intact (AI) and acrosome-reacted (AR) sperm can bind the ZP [21], but only AR sperm reach the perivitelline space and fuse with the oolemma, making this reaction essential. Although the ZP was historically seen as the main trigger, only ~5% of sperm reaching the oocyte in mice are AI [56].

Next, sperm must penetrate the ZP, recognizing it in a species-specific manner. The identity of the ZP molecule acting as the sperm receptor remains debated [11,28,54]. Studies with purified human ZP proteins suggest ZP1, ZP3, and ZP4 bind mainly to AI sperm, while ZP2 prefers AR sperm [11]. In transgenic mice, human sperm bind oocytes expressing ZP2 but not ZP1, ZP3, or ZP4, suggesting a key role for ZP2 [11]. Proteolytic cleavage of ZP2’s N-terminal reduces sperm binding [57], and early domain-swapping studies supported ZP2 as the primary receptor [58]. However, mice expressing ZP2 lacking the N1 domain maintain binding capacity but show poor penetration and severe subfertility [5], casting doubt on ZP2’s receptor role. Alternatively, ZP3, particularly its C-terminal region or associated oligosaccharides, may act as the sperm receptor [54]. Gene-deletion studies remain inconclusive, as ZP2 and ZP3 are both required for ZP formation in mice [59]. How sperm traverse the ZP network remains unclear [54].

After fertilization, the ZP is modified to block polyspermy. The cortical reaction releases enzymes that cleave ZP2, primarily via ovastacin and crosslink ZP3 through transglutaminase 2, increasing ZP rigidity [60]. This “ZP hardening” prevents further sperm penetration and reinforces structural integrity [61]. While ZP3 has been historically thought to trigger the acrosome reaction, most sperm initiate it before contacting the ZP, during transit through the oviduct or cumulus [62,63]. ZP3 primarily maintains ZP integrity, whereas ZP2 mediates post-acrosome-reaction binding, enabling sperm to reach the oolemma [58]. In bovines, de la Fuente et al., [4] showed that in vitro matured (IVM) oocytes, although genomically and cytoplasmically competent, often have porous ZPs. Exposure to OF or in vivo conditions induces conformational changes, incorporating OVGP1 and producing a more homogeneous, less porous structure, which may help prevent polyspermy. While ZP thinning is often attributed to blastomere divisions, proteolytic activity in the female tract also remodels the ZP to facilitate embryo hatching, influenced by asynchronous divisions and maternal factors [4,64]. These structural and functional changes are essential for hatching and implantation.

### 3.4. Journey Through the Oviduct

In animals with internal fertilization, the oviduct plays a central role by providing a specialized environment that supports gamete transport, final maturation, fertilization, and early embryonic development. Connecting the ovaries to the uterus, the oviduct comprises four anatomical regions: the infundibulum, ampulla, isthmus, and uterine-tubal junction. During ovulation, metaphase II oocytes surrounded by expanded cumulus cells are released into the infundibulum [22]. As they transit to the ampulla, oocytes undergo physiological modifications, notably within the ZP, the glycoprotein-rich extracellular matrix surrounding the oocyte [65].

Several studies emphasize the role of the ampullary region in facilitating both fertilization and ZP maturation, a process commonly termed ZP hardening. This modification occurs in two sequential phases: the first within the dominant follicle, and the second during the oocyte’s passage through the ampulla [22,66,67]. Coy et al. [52] introduced the concept of pre-fertilization ZP hardening, distinct from the post-fertilization cortical reaction. Oviduct-mediated ZP hardening is conserved across ungulates and complements oocyte-derived responses. In vitro co-culture with ampullary epithelial cells enhances ZP hardening, reduces polyspermy, and improves fertilization. Conditioned media from these cells, particularly when derived from direct interactions with cumulus-oocyte complexes, further support ZP hardening and early embryonic development [68,69].

Oviductal secretory cells produce high molecular weight glycoproteins, termed oviduct-specific glycoproteins (OGPs), which bind the ZP and perivitelline space during oocyte transit, with some persisting on embryos until implantation. Porcine IVF studies show that OVGP1 and peri-ovulatory OF promote ZP hardening and monospermy [65]. Cross-species studies using bovine OF reveal additional factors, including heat shock proteins (HSPs), protein disulfide isomerases (PDIs), and heparin-like glycosaminoglycans (GAGs), which collectively enhance ZP resistance to enzymatic digestion and sperm penetration. These findings illustrate that the oviduct provides a hormonally regulated, species-specific microenvironment actively shaping ZP maturation and fertilization competence [51].

Recent studies show that OVGP1 also remodels ZP architecture during oviductal transit. The C-terminal region of OVGP1 penetrates the ZP, creating a porous, net-like structure typical of fertilization-competent oocytes, which enhances resistance to proteolytic digestion and promotes monospermy. Truncated OVGP1 lacking this domain fails to remodel the ZP effectively [64]. OVGP1 is also endocytosed by the oocyte, suggesting roles in oocyte–ZP communication and in early development. Immunolocalization studies detect OVGP1 in the microvilli of zygotes, the perivitelline space, and early embryo multivesicular bodies [70].

Although OVGP1 is critical in some species, knockout and pseudogene studies indicate it is not essential for fertilization in all species [19,71]. Its consistent in vivo and in vitro expression, including in bovine biomimetic systems [72], highlights its physiological relevance, where functional. This highlights the species-specific and redundant nature of oviductal regulation of ZP maturation [73]. In addition to soluble glycoproteins, extracellular vesicles (EVs) from oviductal epithelial cells carry proteins, lipids, and RNAs that traverse the ZP and deliver bioactive cargo, including OVGP1, into the ooplasm [74,75]. EVs from cumulus cells similarly penetrate the ZP during in vitro maturation, indicating a conserved intercellular communication mechanism [76]. These findings reinforce the concept of the ZP as a dynamic barrier facilitating selective molecular exchange during oviductal maturation and fertilization. The oviduct also supports early embryo development and transport, guiding fertilized oocytes to the uterine cavity. It provides growth factor-rich secretions, protects embryos from oxidative stress, and mediates the first embryo–maternal interactions [22,61,73,74]. Transport likely involves ciliary beating, muscular contractions, and fluid flow [77].

During the oocyte-to-embryo transition, the ZP acts as a protective barrier against mechanical stress and enzymatic degradation. This barrier maintains structural integrity until blastocyst hatching [28]. ZP elasticity may serve as an indicator of embryo quality [78]. In vivo-derived porcine embryos exhibit more digestion-resistant ZP than in vitro counterparts, highlighting the role of oviduct-specific modifications [79]. Knockouts of ZP1 in mice or ZP4 in rabbits cause structural fragility, impaired transport, and developmental arrest, underscoring the ZP’s mechanical role in embryo survival [59,80]. Finally, the ZP facilitates smooth embryo transport by maintaining spherical shape and reducing adhesion to the oviductal epithelium [77,81]. Together, these observations highlight the dynamic crosstalk between the oviduct, oocyte, and early embryo, emphasizing the importance of replicating this microenvironment in ARTs.

### 3.5. Role in Oocyte Fertilization, Embryo Protection, and Embryo Implantation

**Figure 4:**
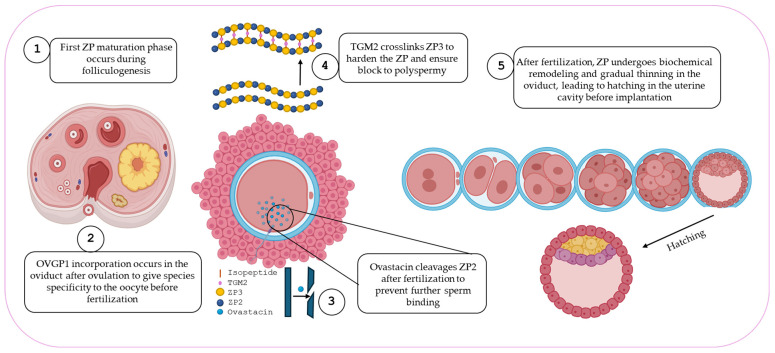
Schematic summary of ZP maturation from ovary to implantation.

During fertilization, the fusion of two highly differentiated haploid gametes is mediated by the ZP, which orchestrates three critical processes: species-specific sperm recognition (Figure 4), sperm binding to the oocyte, and prevention of polyspermy. Fusion of the sperm membrane with the oolemma triggers a cascade of structural and biochemical changes, including ZP hardening. These modifications prevent polyspermy and the formation of nonviable polyploid embryos. One such mechanism is the cortical reaction, where cortical granules release their contents post-fertilization, causing the ZP matrix to lose its capacity to bind additional sperm [1,82].

A key component of this process is the cleavage of ZP2 glycoprotein. This cleavage is mediated by ovastacin, a cortical granule metalloendoprotease encoded by Astl. Ovastacin targets the N-terminal domain of ZP2, and proteolytically degrades it, thereby preventing further sperm interaction. Functional evidence comes from Astl knockout mouse models, in which ZP2 remains uncleaved after fertilization. Consequently, sperm continue to bind the ZP of fertilized eggs, confirming that ovastacin-mediated ZP2 cleavage is essential for establishing a definitive block to polyspermy [83]. Supporting this, Maddirevula et al. [84] reported that adult women homozygous for a non-functional variant of ovastacin exhibit markedly reduced fertility.

Nishio et al. [5] further demonstrated that, following fertilization, ovastacin cleaves the N-terminal region of ZP2, triggering its oligomerization. This rearrangement results in extensive cross-linking between ZP filaments, reinforcing the structural integrity of the egg coat. The ZP is composed of a left-handed double-helical architecture formed by interwoven type I and type II protofilaments. Upon cleavage, liberated ZP2 fragments self-assemble, stabilizing the matrix, promoting filament compaction, and preventing polyspermy. Fetuin-B is acknowledged as an endogenous inhibitor of ovastacin [5]. Moreover, Von Wiegen et al. [85] showed that disruption of the ovastacin–fetuin-B balance alters ZP2 cleavage timing, causing premature zona hardening and reduced fertilization efficiency. Together, these findings underscore the critical importance of precise regulation of ZP2 proteolysis for ZP remodeling and reproductive success.

Transgenic mice with defective ZP2 cleavage (ZP2^mut) show that sperm continues to bind to the zona after fertilization, although monospermy is maintained. This suggests that ZP2 cleavage is essential for preventing post-fertilization sperm binding but not strictly required to block polyspermy [58]. ZP3 also undergoes rapid post-fertilization modifications, likely induced by cortical granule enzymes, which render it unrecognizable to sperm. Additionally, ZP3 also undergoes covalent cross-linking catalyzed by transglutaminase 2 (TGM2). This calcium-dependent enzyme, secreted from the oocyte cortex via a SNARE-regulated pathway, reinforces the ZP by forming isopeptide bonds between glutamine and lysine residues. This increases matrix rigidity, and limits further sperm penetration. Biochemical and functional assays in murine and porcine models identify ZP3 as the sole zona substrate of TGM2. Pharmacological inhibition or genetic ablation of TGM2 increases polyspermic fertilization, evidenced by elevated rates of polypronuclear zygotes and decreased zona resistance to proteolytic degradation. Conversely, recombinant TGM2 supplementation restores ZP integrity and reduces polyspermy in a concentration-dependent manner [60,86].

These post-fertilization biochemical alterations in ZP2 and ZP3, together with modifications of the ZP matrix and oolemma [5,28,57], establish a robust block to polyspermy. Mouse models have been instrumental in elucidating ZP glycoprotein functions. ZP3 acts not only as a structural component, but also as a signaling molecule. In ZP3-deficient mice, no two-cell embryos were recovered after mating, indicating fertilization or early development failure [59]. Similarly, embryos with enzymatically removed ZPs cannot progress through the oviduct and highlighting the ZP’s crucial structural role in embryo protection and transport.

Beyond fertilization, the ZP continues to play a vital role until implantation. It forms a protective shell around the preimplantation embryo, shielding it mechanically and preventing premature interactions with the maternal reproductive tract. In rabbits, embryos lacking ZP4 failed to develop to the blastocyst stage [83]. The intact ZP guides the embryo from the oviduct to the uterus, preventing ectopic implantation by separating the embryo from the oviductal epithelium [87]. Its glycoprotein-rich ZP protects the embryo from mechanical stress and enzymatic degradation [88,89]. In addition, it may exert immunomodulatory effects, protecting the embryo from maternal immune recognition [90]. As the embryo reaches the blastocyst stage, the ZP undergoes thinning and degradation until the hatching process, which is essential for successful implantation. Hatching is influenced by three main factors: mechanical expansion of the blastocyst, enzymatic ZP degradation by hydrolases secreted by trophectoderm cells, and proteases from uterine fluids that weaken the ZP [91].

### 3.6. Specificity of Sperm Binding

Successful mammalian fertilization involves a tightly coordinated sequence: sperm capacitation, acrosome reaction, species-specific sperm-egg recognition, penetration of the ZP, and membrane fusion with the oocyte. Species-specific gamete recognition ensures reproductive success by promoting genetic compatibility and maintaining species integrity. Despite its fundamental role, the molecular basis of sperm-oocyte interaction, particularly the species-specific features, remains partially understood. Evidence indicates that species specificity operates at multiple levels, from initial sperm-egg attraction to gamete membrane fusion [27].

Interspecies differences in binding affinities help prevent cross-species fertilization, as seen with reduced interaction between human JUNO and mouse IZUMO1 [92]. While the IZUMO1–JUNO interaction contributes to species specificity, ZP glycoproteins directly prevent heterologous fertilization. Genetic studies reveal that sperm-ZP binding is primarily mediated by the N-terminal domain of ZP2. Targeted mutagenesis of canonical O-glycosylation sites in mouse ZP3 (Ser^332 and Ser^334) does not impair fertilization, suggesting these carbohydrate moieties are dispensable in rodents [93].

In contrast, in bovine and porcine species, sialylated glycans are crucial for sperm binding and penetration. Terminal sialic acids, particularly α2-3-linked Neu5Ac and Neu5Gc, mediate this interaction. Their enzymatic removal significantly reduces sperm adhesion and fertilization [4]. These findings highlight a dual role for sialic acids in bovine ZP, supporting both nonspecific binding and species-specific recognition, modulated by the sialylation of ZP and OVGP1.

Species-specific fertilization barriers arise from differences in ZP architecture and glycosylation. Human ZP contains sialyl-Lewis^x epitopes absent in murine ZP. However, many non-human mammals incorporate Neu5Gc, which is not found in humans due to CMAH inactivation [94]. These differences provide glycan-level cues for taxon-specific binding, although heterologous fertilization can still occur experimentally [4], suggesting that both ZP structure and glycosylation jointly modulate species specificity.

However, quantitative data on the glycan composition of the human ZP remain limited. Comprehensive glycomic profiling is still lacking, and most available evidence derives from animal models. Further characterization is needed to define the precise distribution and abundance of these glycans in the human ZP. Such information is essential to understand how glycosylation regulates sperm–oocyte interactions and could inform the design of biomimetic or glycan-optimized strategies to improve the efficiency of ART.

Also, extrinsic modulation by the oviductal environment further shapes ZP function. OF induces compositional and structural ZP changes that enhance oocyte maturation and sperm recognition [22,51]. OVGP1, an oviductal glycoprotein, is critical for species-specific fertilization. Only homologous OVGP1 enables sperm binding and penetration, while heterologous OVGP1 or absence of OVGP1 diminishes fertilization efficiency across multiple species, including hamsters, cows, pigs, and humans [4]. OVGP1 penetrates the ZP via its conserved C-terminal region, modifying glycosylation and coating ZP pores with sialic acid-rich oligosaccharides recognized in a species-specific manner. Scanning electron microscopy confirms uniform ZP coating only with homologous OVGP1, whereas heterologous protein or absence of OVGP1 results in incomplete coverage [4,95]. Thus, OVGP1 not only enhances fertilization efficiency but also reinforces species-specific sperm recognition, highlighting a cooperative mechanism in which intrinsic ZP properties and extrinsic oviductal factors jointly determine homologous fertilization.

### 3.7. Assisted Hatching: Rationale, Technique, and Clinical Outcomes

ZP thickness in early-stage embryos is strongly associated with implantation success. Comparative analyses have shown that embryos surrounded by zonae pellucidae with inadequate biomechanical properties exhibit reduced implantation potential, thereby reinforcing the concept that the zona pellucida fulfils a dual function in mediating gamete interaction and supporting subsequent embryonic development [96]. Implantation rates have also been correlated with ZP-related events such as birefringence and spontaneous hatching [97].

During in vitro culture, human embryos typically show gradual ZP thinning, whereas zygotes that fail to cleave demonstrate minimal change in ZP thickness [96,98]. Abnormalities in ZP structure or blastocyst hatching are linked to implantation failure and early pregnancy loss [91]. After embryo transfer, roughly 30% of embryos fail to implant, with failure rates rising to nearly 70% for blastocysts that do not undergo spontaneous hatching [97,99]. These observations underscore the critical importance of hatching for successful implantation and pregnancy [100]. Indeed, thawed blastocysts that re-expand or reach the full hatching stage show higher pregnancy rates, underscoring the prognostic value of these morphological dynamics [101,102,103]. In vitro-cultured blastocysts often exhibit increased ZP hardening compared to their in vivo counterparts, impairing hatching and subsequent implantation [104].

Natural hatching is regulated by both physical and molecular factors. Physical factors include blastocyst expansion and ZP rupture, whereas molecular regulation involves multiple genes. Human hatched blastocysts show higher mRNA expression of CTSV, GATA3, and CGB compared to non-hatched counterparts [105]. Furthermore, dynamic expression of the Notch gene family suggests that Notch signaling plays a crucial role in mouse hatching progression, and disruptions in this pathway can impair the process, highlighting its importance in early embryonic development [106].

In this context, assisted hatching has proven effective in enhancing implantation rates in both mouse [105] and human embryos [106]. However, the clinical evidence in humans remains far from conclusive. Previous researchers have reported inconsistent outcomes, with some other studies showing no significant improvement in live birth rates [107]. Factors such as maternal age, the use of fresh versus thawed embryos, history of implantation failure, or preimplantation genetic testing analysis (PGT-A) strongly influence outcomes. Moreover, individual IVF laboratories often apply their own criteria and employ distinct techniques, including mechanical, chemical, or laser-assisted hatching, adding further variability to the evidence base.

Given these inconsistencies, recent guidelines emphasize that assisted hatching should not be used routinely. Instead, it may be considered only for well-defined patient groups [108]. To determine its true clinical value, future research should include large, well-stratified randomized controlled trials (RCTs) that precisely define patient characteristics, standardize procedural parameters, and confirm whether modest improvements in implantation genuinely translate into higher live birth rates.

## 4. Clinical Implications and Future Perspectives

The ZP is a complex glycoprotein matrix whose absence or structural defects represent a major cause of infertility in both animal models and humans. This may result from abnormalities in glycoprotein composition or receptor function during key events such as sperm recognition, the acrosome reaction, and uterine implantation [30,31,34,35,36]. Just as assisted hatching may represent a major advancement in ARTs by facilitating ZP rupture and improving implantation [109], emerging research is exploring novel strategies targeting ZP structure and function to treat infertility and develop non-hormonal contraceptives.

Mutations in genes such as hZP4 can lead to an abnormally thin, discontinuous, and structurally deficient ZP, severely impairing sperm–oocyte interaction and reducing fertilization rates [37]. In a rabbit model, CRISPR/Cas9-mediated inactivation of ZP4 produced a thinner, unstable ZP and complete infertility, highlighting the essential structural role of ZP4 [80]. Partial weakening of the ZP preserved blastocyst development and editing efficiency, whereas complete removal increased mutation rates but compromised embryo viability. These findings illustrate the delicate balance between genome editing efficiency and embryonic health, emphasizing the importance of modulating ZP conditions during experimental manipulations.

Pathogenic variants in ZP1, ZP2, and ZP3 have been identified in women with genuine empty follicle syndrome (GEFS), a rare disorder in which oocytes degenerate despite normal ovarian stimulation [110,111]. These mutations disrupt ZP protein secretion and assembly, leading to infertility. CRISPR-based models provide valuable platforms to study such mutations, enhancing both diagnostics and the development of targeted therapies. Interestingly, not all oocyte-specific genes are essential for fertility: knockout of Oosp1, Oosp2, and Oosp3 in mice did not impair female fertility, highlighting the importance of in vivo functional screens to distinguish essential from dispensable reproductive genes [112]. Overall, CRISPR/Cas9 technologies are proving invaluable not only for precise gene editing but also for functional characterization of reproductive genes, informing which molecular pathways are genuinely critical for female reproductive success. The clinical translation of these approaches requires careful ethical consideration regarding safety, equitable access, and broader societal implications.

It has been recently demonstrated that OVGP1 can structurally modify the ZP to prevent polyspermy and cross-species sperm entry [4]. Specifically, bovine ZPs devoid of cytoplasm and unexposed to OF were penetrable by heterologous sperm, including human, while exposure to OVGP1 restored species-specificity. The use of bovine ZPs may enable more precise sperm selection in ICSI cycles by evaluating functional parameters such as the acrosome reaction, DNA integrity, and blastocyst developmental competence.

Nevertheless, the translation of these results into clinical practice must proceed with caution. Further studies are essential to confirm the efficacy of these molecules in human IVF and to ensure their safety across multiple dimensions, including potential immunogenicity, epigenetic modifications, and alterations in the omic profiles of developing embryos. Rigorous preclinical testing and well-designed clinical trials will be necessary to validate these approaches before any implementation in ART protocols. This evidence will be crucial to ensure that the use of OVGP1 and EVs enhances embryo quality and implantation potential without compromising long-term embryonic or offspring health.

Detailed understanding of ZP structure and function also enables the development of non-hormonal contraceptives. ZP-based vaccines have been widely explored to induce immunological infertility. For example, a monoclonal antibody (IE-3) or its scFv fragment targeting a defined N-terminal region of ZP2 has been shown to effectively block sperm binding and fertilization in mice without inducing ovarian damage or impairing follicular maturation [113].This peptide-based approach was effective in mouse models, and transgenic assays demonstrated that the human homolog competitively inhibited human sperm binding to humanized mouse oocytes, supporting its potential as a species-specific, non-hormonal contraceptive [114]. In dogs, native or recombinant ZP glycoproteins effectively inhibited fertility, representing a promising strategy for ethical wildlife population management [82]. Furthermore, targeting sperm proteins that interact with the ZP opens additional avenues for reversible, non-hormonal contraceptive strategies by competitive inhibition of sperm adhesion and penetration [38].

However, some findings from animal studies reveal that ZP-based non-hormonal contraceptives can effectively prevent fertilization, but results regarding reversibility, ovarian safety, and immune effects remain inconsistent. Other studies, particularly those involving ZP3-derived vaccines, have reported follicular depletion, oocyte loss, and ovarian inflammation associated with stronger or less specific immune responses [115]. Research in wildlife using porcine ZP formulations shows a similar pattern: fertility returned in some females once antibodies waned, while others experienced prolonged infertility or histological changes in the ovaries [116]. These contrasting findings suggest that immunogenicity, species specificity, and vaccine formulation exert a decisive influence on both efficacy and safety.

In conclusion, manipulation of the ZP represents a promising frontier in reproductive medicine, with potential applications in personalized infertility treatment, improved IVF outcomes, and the development of next-generation non-hormonal contraceptives. However, future studies will be essential to confirm the reversibility, immunogenicity, species specificity, and impact on ovarian reserve of these approaches. Ensuring safety through standardized experimental designs and long-term monitoring will be critical before any clinical implementation of ZP-based contraceptive strategies can be considered.

## Figures and Tables

**Table 1:** Proteins present in the zona pellucida (ZP) of different mammalian groups. In eutherian mammals, ZP3-1c is usually referred to as ZP3.

Group of Mammals	ZP1	ZP2	ZP3-1a	ZP3-1b	ZP3-1c	ZP3-2	ZP4	ZPAX	ZPY	References
*Mus* *musculus*	✓	✓			✓					[13,14,15]
Most eutherian mammals	✓	✓			✓		✓			[6,14]
Dog, cow, sow, dolphin, fox		✓			✓		✓			[14,16,17]
American marsupials	✓	✓		✓	✓					[14,18,19]
Australian marsupials	✓	✓	✓	✓	✓		✓	✓		[14,20]
Monotremes	✓	✓	✓	✓		✓	✓	✓	✓	[14,21]

**Table 2:** (Adapted from [48]). Average and variance values of ZP thickness and retardance measured in individual human oocytes and embryos. Values are presented as mean ± SD (one-tailed *t*-test; *p* < 0.05). ^a^ Statistically significant difference between immature and mature oocytes. ^b^ Statistically significant decrease between mature oocytes and embryos. ^c^ Statistically significant increase between mature oocytes and embryos.

	Immature Oocytes	Mature Oocytes	Day 3 Embryos
**Layer 1**			
Thickness (µm)	10.5 ± 2.3	9.8 ± 2.1 ^b^	7.9 ± 1.9
Retardance (nm)	3.26 ± 1.27	2.84 ± 1.07	3.00 ± 0.86
**Layer 2**			
Thickness (µm)	3.4 ± 0.6	3.7 ± 0.9	3.6 ± 1.2
Retardance (nm)	0.21 ± 0.07 ^a^	0.24 ± 0.05 ^c^	0.33 ± 0.29
**Layer 3**			
Thickness (µm)	6.5 ± 2.1	6.1 ± 1.7 ^b^	3.7 ± 1.4
Retardance (nm)	0.91 ± 0.24	0.95 ± 0.23 ^b^	0.78 ± 0.27
**Total Zona**			
Thickness (µm)	20.4 ± 2.4	19.5 ± 2.2 ^b^	15.2 ± 2.8

## Abbreviations

The following abbreviations are used in this manuscript:

AI	Acrosome-intact
AR	Acrosome-reacted
ARTs	Assisted Reproductive Techniques
EVs	Extracellular vesicles
GAGs	Heparin-like glycosaminoglycans
GEFs	Genuine empty follicle syndrome
HSPs	Heat shock proteins
IVM	in vitro matured
OF	Oviductal fluid
OVGP1	Oviductal glycoprotein 1
NTR	N-terminal region
PGT-A	Pre-implantation genetic testing analysis
RCTs	Randomised controlled trials
TGM2	Transglutaminase 2
ZP	Zona pellucida
